# LC‐HRMS Profiling and Physicochemical Characterization of Strawberries From Agroclimatic Region in Java, Indonesia

**DOI:** 10.1002/cbdv.71832

**Published:** 2026-10-21

**Authors:** Soraya Habibi, Einstivina Nuryandani, Nanik Suhartatik, Fadilah Husnun, Akhmad Mustofa, Nurika Yepi Prasetyaningtyas, Titiek Farianti Djaafar, Tri Marwati, Anna Fajariyah, Ulyatu Fitrotin

**Affiliations:** ^1^ Department of Biology Faculty of Science and Technology Universitas Terbuka Tangerang Selatan Indonesia; ^2^ Department of Food Technology Faculty of Science and Technology Universitas Slamet Riyadi Surakarta, Jawa Tengah Indonesia; ^3^ Research Center for Technology and Food Processing National Research and Innovation Agency (BRIN) Gunungkidul Yogyakarta Indonesia

**Keywords:** agroclimatic variation, anthocyanin, antioxidant, LC‐HRMS, strawberry

## Abstract

Strawberries are valued for their nutritional and bioactive compounds, yet consumer acceptance in Indonesia remains limited due to high acidity and variable sweetness. This study evaluated the influence of agroclimatic variation across four highland regions of Java, Indonesia (Batu, Ciwidey, Purbalingga, and Tawangmangu) on the physicochemical and bioactive composition of strawberries using soil fertility assessment, antioxidant assays, and LC‐HRMS (Liquid Chromatography‐High Resolution Mass Spectrometry) analysis. Significant differences (*ρ* < 0.05) were observed among regions: Batu exhibited the highest total anthocyanin content (TAC) (18.15 ± 1.11 mg cyanidin‐3‐O‐glucoside/100 g) and total soluble solids (TSS) (8.90 ± 0.10°Brix), while Ciwidey and Batu showed elevated sugar levels (2.15 ± 0.04% and 2.17 ± 0.31%). TAC was greatest in the Batu, corresponding with its intense pigmentation. Strawberries from Tawangmangu contained diverse bioactive profile, including ellagic acid, kaempferol, GABA (Gamma amynobutyric acid), and miquelianin with the highest total phenolic content (TPC) (7.41 ± 1.20 mg gallic acid equivalent (GAE)/100 g). Antioxidant activity was strongest in Batu and Ciwidey (DPPH‐ 2,2‐diphenyl‐1‐picrylhydrazyl scavenging >95%). These findings suggest that altitude, temperature, and soil mineral composition were associated with the strawberry quality, providing a scientific basis for region‐specific cultivation and the development of functional food in tropical highland systems.

## Graphical abstract

Agroclimatic variation may influence strawberry sugar, acidity, and antioxidant levels. Cooler and wetter climates are associated with enhanced accumulation of bioactive compounds in strawberries. LC‐HRMS analysis reveals region‐specific metabolite fingerprints, highlighting the molecular diversity shaped by environmental conditions. The integrated visualization illustrates how agroclimatic factors, soil composition, and metabolite profiles collectively determine fruit quality and support region‐specific cultivation strategies.

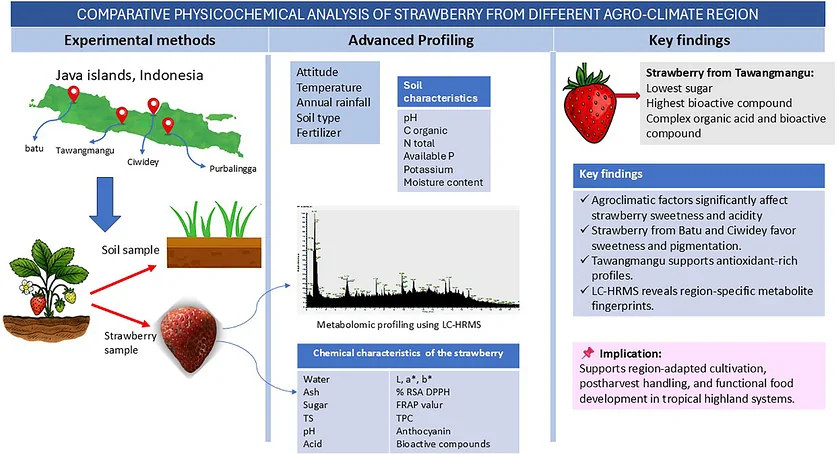

## 1 Introduction

Strawberry (*Fragaria × ananassa*) is globally recognized as a high‐value horticultural commodity because of its rich nutritional profile, appealing sensory qualities, and diverse health‐promoting bioactive compounds. It is considered a functional food due to the presence of bioactive constituents with various physiological effects [1]. Strawberries contain diverse bioactive compounds, including vitamins, polyphenols, organic acids, ellagitannins, anthocyanins, dietary fiber, and essential minerals. Within these groups, flavonoids such as ellagic acid, kaempferol, and quercetin, together with phenolic acids and glycosides, are particularly relevant because they contribute to antioxidant and anti‐inflammatory properties [2, 3, 4, 5, 6, 7, 8]. Importantly, the concentration of anthocyanins and phenolic acids is highly responsive to agroclimatic variation, making them critical markers for evaluating fruit quality in different cultivation regions. This highlights the dual nutritional and ecological significance of strawberry bioactive in the context of tropical highland production

In Indonesia, strawberry cultivation is concentrated in highland areas, notably in Lembang and Ciwidey (Bandung), Tawangmangu (Surakarta), Magelang, Wonosobo, Batu (East Java), and Purbalingga, which are recognized as key production centers. Strawberries from these regions typically have high acidity, which strongly influences consumer preferences [8, 9]. Acceptance is largely determined by the sugar‐to‐acid ratio in the fruit [10]. Citric acid is the predominant organic acid in strawberries, with malic acid and other acids contributing significantly to their flavor profile [6].

Recent studies have highlighted the significant influence of agroclimatic conditions such as altitude, temperature, and soil composition on the nutritional and phytochemical profiles of berry fruits, including strawberries [8, 9, 11]. Akter et al. [12], demonstrated that mulching significantly improved root development, leaf biomass, and fruit yield, with genotypes such as Rabi 3 showing superior performance in terms of growth, yield, and biochemical parameters, while Sweet Charlie exhibited the highest antioxidant activity. In contrast, Prihandarini et al. [13], found that combining goat manure, compost, and Rhizobium Microorganism (RIM) biofertilizer enhanced vegetative growth (plant length and leaf number) and increased fruit production by up to 45.2%, while also improving sweetness levels (°Brix). These findings suggest that both mulching and organic fertilization strategies play complementary roles: mulching optimizes microclimatic and soil physical conditions, whereas organic amendments enrich soil fertility and microbial activity [12, 13, 14].

In Indonesia, strawberry cultivation spans diverse highland regions, yet systematic studies linking agroclimatic variation to the accumulation of specific bioactive compounds remain scarce. This gap limits the understanding of how altitude, temperature, and soil fertility shape nutritional quality, thereby constraining efforts to optimize cultivation practices, enhance postharvest handling, and develop functional food products tailored to regional conditions. However, studies examining the influence of growing environmental conditions on the bioactive compound and quality of strawberries through chemical composition analysis have not yet been conducted.

This study evaluates the influence of agroclimatic conditions on strawberry quality across four cultivation regions in Java, Indonesia (Tawangmangu, Purbalingga, Ciwidey–Bandung, and Batu–East Java). The research aims to characterize nutrient composition and bioactive compound and to determine how agroclimatic variation shapes these attributes. To date, no systematic study has applied LC‐HRMS to profile strawberries from diverse Indonesian highland regions. Previous work has focused mainly on agronomic traits or sensory evaluation, leaving a gap in understanding the molecular composition of strawberries under tropical highland conditions. By providing the first comparative LC‐HRMS profiling, this study identifies key compounds such as flavonoids, organic acids, and phenolics, and highlights their variation across regions. The findings establish a scientific basis for linking agroclimatic variation with fruit quality, offering insights for postharvest processing, functional food development, and region‐specific cultivation strategies.

## 2 Results and Discussion

### 2.1 Soil Characteristics and Agroclimatic Regions

Integrated data on agroclimatic and soil parameters revealed distinct environmental profiles among the four strawberry cultivation sites (Table 1). Tawangmangu, located at the highest altitude (1800 m), exhibited the coolest average temperature (15–18°C) and the highest annual rainfall (∼3 500 mm), resulting in the greatest soil moisture content (55.20 ± 0.21%). These conditions were associated with an environment that may favor microbial activity and organic matter accumulation, as reflected in elevated organic carbon (8.63 ± 0.01 ppm) and total nitrogen (0.56 ± 0.03%) levels. However, direct measurements of microbial dynamics were not conducted in this study, and the observed association should be interpreted with caution. In contrast, Batu, situated at a lower elevation (1200 m) with warmer temperatures (20–22°C) and lower rainfall (∼2500 mm), showed the lowest moisture content (26.66 ± 0.15%) but the highest available phosphorus (40.21 ± 0.16 ppm) and exchangeable potassium (0.37 ± 0.02 meq per 100 g). These soil nutrient conditions are associated with fruit quality attributes such as sweetness and firmness [15, 16, 17], although such traits are likely influenced by multiple interacting agroclimatic and soil factors rather than a single determinant.

**TABLE 1:** Agroclimatic characteristics of strawberry cultivation sites in Java, Indonesia.

Agroclimatic Characteristics	Location			
	Purbalingga	Tawangmangu	Ciwidey	Batu
Agroclimatic condition				
Altitude (m asl)	1300	1800	1700	1200
Longitude	−7.23984252, 109.28807547	−7.66586989, 111.18144208	−7.11351342, 107.44312952	−7.86862838, 112.54181456
Average temperature (°C)	19–21	15–18	18–20	20–22
Annual rainfall (mm)	∼2800	∼3500	∼3000	∼3000
Soil type	Sandy clay loam	Volcanic loam	Volcanic loam	Andosol (volcanic)
Fertilizer used	Cattle manure	Cattle manure	Cattle manure	Cattle manure

Soil characteristics				
pH	7.41 ± 0.00^d^	6.78 ± 0.00^b^	7.13 ± 0.01^c^	6.60 ± 0.01^a^
C organic, ppm	3.05 ± 0.00^a^	8.63 ± 0.01^d^	3.08 ± 0.01^b^	4.96 ± 0.00^c^
N total (%)	0.46 ± 0.04^b^	0.56 ± 0.03^c^	0.45 ± 0.03^b^	0.35 ± 0.01^a^
Available phosphorus, ppm	34.29 ± 0.12^b^	30.14 ± 0.08^a^	35.73 ± 0.12^c^	40.21 ± 0.16^d^
Exchangeable potassium (meq/100 g soil)	0.32 ± 0.04^a^	0.30 ± 0.00^a^	0.32 ± 0.02^a^	0.37 ± 0.02^b^
Moisture content (%)	34.75 ± 0.73^b^	55.20 ± 0.21^d^	45.05 ± 0.08^c^	26.66 ± 0.15^a^

*Note*: Values with the same notation (a. b. c, d) in the same row were not significantly different (*p* < 0.05) based on the results of the One‐Way ANOVA analysis.

Ciwidey and Purbalingga exhibited intermediate profiles. Ciwidey had moderate moisture content (45.05 ± 0.08%) and balanced nutrient levels, while Purbalingga had the highest soil pH (7.41 ± 0.01) and relatively low organic carbon (3.05 ± 0.01 ppm). These variations in agroclimatic and soil conditions are expected to associated with the biosynthesis of bioactive compounds in strawberry fruits, particularly phenolics, anthocyanins, and organic acids. The data indicate that Tawangmangu provides a favorable environment for antioxidant accumulation due to its cooler, wetter climate and higher organic content. Batu may support higher sugar levels through mineral‐driven enhancement.

The findings of this study align with and extend recent research on the effects of agroclimatic conditions and organic fertilization on strawberry quality. Ecological variation associated with the biosynthesis of bioactive compounds in strawberries, particularly flavonoids and anthocyanins, through transcriptomic and metabolomic analysis [18]. Consistent with previous findings, strawberries grown in Tawangmangu, characterized by cooler temperatures and higher rainfall, showed the highest levels of organic carbon, nitrogen, and soil moisture. These conditions were associated with elevated antioxidant activity and TPC. Similarly, manure‐based fertilization enhances vitamin C and phenolic content, supporting the observation that manure practices across all sites contributed to the baseline of bioactive compounds [19].

### 2.2 Chemical Characteristics of the Strawberry From Different Regions

#### 2.2.1 Ash and Moisture Content

The ash content of strawberry samples across all regions ranged from 0.17 ± 0.07% to 0.21 ± 0.01%, indicating relatively stable mineral composition despite agroclimatic variation. Strawberries cultivated under organic fertilization in subtropical highlands maintained consistent ash levels between 0.18% and 0.22%. While manure‐based inputs may have contributed to mineral stability under the conditions studied, ash content buffering is more likely associated with the plant's selective nutrient translocation mechanisms, and should be interpreted as an observation specific to these environments rather than a general effect [20]. The moisture content of strawberries in this study ranged from 90.96% to 92.92%, consistent with the results of El‐Hawary et al. [21], who reported moisture levels between 91.0% and 92.5% in Egyptian‐grown cultivars. Similarly, the ash content observed across all agroclimatic regions (0.17–0.21%) corresponds to the reported values (0.18–0.22%), indicating that the mineral composition of strawberries remains relatively stable despite environmental variation.

Moisture content (Table 2), however, exhibited more pronounced variation, ranging from 90.96 ± 0.30% in Batu to 92.92 ± 0.38% in Tawangmangu. These differences reflect the influence of altitude, temperature, and rainfall on water retention in fruit tissues. Adequate soil moisture enhances strawberry growth and reduces the occurrence of hollow fruits, while excessive moisture may dilute soluble solids and diminish flavor intensity [22]. In this study, higher moisture levels in Tawangmangu and Ciwidey were associated with cooler climates and with higher rainfall, potentially contributing to softer texture and increased antioxidant accumulation. Conversely, Batu's lower moisture content may favor the concentration of sugars and anthocyanins, thereby enhancing sweetness and color intensity.

**TABLE 2:** Physicochemical characteristics of Strawberries.

Parameter	Agroclimatic conditions based on the area			
	Purbalingga	Tawangmangu	Ciwidey	Batu
Moisture content (%)	91.22 ± 1.07^a^	92.92 ± 0.38^b^	92.37 ± 1.00^ab^	90.96 ± 0.30^a^
Ash content (%)	0.20 ± 0.08^a^	0.21 ± 0.01^a^	0.17 ± 0.07^a^	0.19 ± 0.03^a^
Total sugar (%)	1.26 ± 0.10^a^	1.43± 0.28^a^	2.15 ± 0.04^b^	2.17 ± 0.31^b^
Total soluble solids (°Brix)	5.10 ± 0.17^a^	7.13 ± 0.21^c^	6.77 ± 0.25^b^	8.90 ± 0.10^d^
Titratable acid (%)	2.84 ± 1.18^a^	4.39 ± 0.90^ab^	5.68 ± 0.90^b^	4.91 ± 1.61^ab^
pH value	3.58 ± 0.07^a^	4.12 ± 0.07^b^	4.58 ± 0.06^c^	4.82 ± 0.07^d^
Fats (%)	0.89 ± 0.06^a^	1.08 ± 0.06^a^	1.12 ± 0.30^a^	0.72 ± 0.04^a^
Protein (%)	0.85 ± 0.02^a^	0.83 ± 0.00^a^	0.74 ± 0.07^a^	0.84 ± 0.00^a^
Crude fiber (%)	0.01 ± 0.00^b^	ND	ND	0.03 ± 0.00^c^
L* (Lightness)	25.68 ± 3.33^a^	35.42 ± 0.08^b^	33.71 ± 3.56^b^	27.31 ± 2.60^a^
a*	27.22 ± 1.74^a^	25.10 ± 1.11^a^	28.28 ± 1.36^ab^	31.52 ± 3.18^b^
b*	5.22 ± 1.49^a^	8.13 ± 0.58^b^	11.20 ± 1.96^c^	2.82 ± 1.53^a^
DPPH scavenging activity (%RSA)	81.29 ± 2.26^a^	87.78 ± 1.71^b^	95.94 ± 1.41^c^	96.66 ± 0.16^c^
FRAP value	12.78± 0.12^bc^	12.99± 0.05^c^	12.19± 0.32^a^	12.46± 0.14^ab^
TPC (mg GAE/100 g)	4.36 ± 2.05^a^	7.41 ± 1.20^b^	2.04 ± 1.49^a^	2.61 ± 0.09^a^
TAC (mg of cyanidin‐3‐O‐glucoside/ 100 g)	5.52 ± 2.65^a^	6.39 ± 2.78^a^	9.29 ± 1.60^a^	18.15 ± 1.11^b^

*Note*: Data were expressed as mean ± SD (*n* = 3). Superscript letters (a, b, c, d) indicate significant differences among agroclimatic regions (*p* < 0.05, one‐way ANOVA, Tukey's HSD). ND = not detected

#### 2.2.2 Sugar Content, Total Soluble Solids, and Titratable Acidity

The chemical composition of strawberries varied significantly across the four agroclimatic regions, particularly in terms of sugar content, TSS, and titratable acidity (Table 2). Samples from Batu and Ciwidey exhibited the highest sugar levels (2.17 ± 0.31% and 2.15 ± 0.04%, respectively), suggesting that warmer temperatures and higher potassium availability in these regions may enhance carbohydrate accumulation. This observation is consistent with Nakro et al. [23], who found that potassium fertilization improves sugar translocation and sweetness in strawberries. Potassium contributes to phloem loading and sugar translocation [24], while elevated temperatures can enhance enzymatic activity in carbohydrate synthesis and accumulation [25]. Fruit sweetness, however, is likely determined by multiple interacting agroclimatic and physiological factors rather than a single mechanism. Total soluble solids followed a similar pattern, with Batu showing the highest value (8.90 ± 0.10 °Brix), indicating greater dry matter concentration and potentially stronger flavor intensity. Previous studies have reported TSS (°Brix) ranging from 6.6 to 10.2 °Brix, pH values between 3.4 and 3.7, and titratable acidity values of 0.60–0.70%. These values differ from those observed in the samples, which may reflect variations in cultivar genetics, agroclimatic conditions, harvest maturity, and methodological approaches. Recent reports confirm that strawberry quality traits such as sugar and organic acid profiles vary significantly among cultivars and environments [20, 22, 24], highlighting the importance of considering environmental and genetic factors when comparing fruit quality across studies [26].

In contrast, strawberries from Purbalingga contained the lowest sugar (1.26 ± 0.10%) and TSS (5.10 ± 0.17 °Brix), which could reflect lower potassium levels and moderate soil moisture. Interestingly, Tawangmangu, despite its cooler climate and high soil moisture, produced strawberries with intermediate sugar content (1.43 ± 0.28%) but relatively high acidity (4.39 ± 0.90%). This aligns with the findings of Duan et al. [27], who reported that cooler environments enhance organic acid biosynthesis, contributing to greater tartness and antioxidant potential. Overall, the results suggested that agroclimatic conditions and soil mineral composition are associated with variation in the balance between sweetness and acidity, which is an important factor for consumer preference and functional food development.

#### 2.2.3 Fats, Protein, and Crude Fiber

The fat content of strawberries from four different regions ranged from 0.72 to 1.12%, while the protein varied between 0.74% and 0.85% (Table 2). Statistically, there is no significant difference, as the p value is greater than 0.05. Overall, the protein in strawberries was relatively low across all samples. According to Newerli‐Guz et al. [6], the protein present in strawberries includes allergen proteins such as Fra a and Fra a 2, which are associated with pigment production in the fruit. These proteins are commonly found in both unripe and ripe strawberries, with their concentrations decreasing as the fruit matures. Total soluble protein found in strawberries reaches 0.85%. The fat in strawberries is primarily composed of essential oils, which are responsible for their aroma [28]. Unsaturated fatty acids found in strawberries include palmitic acid, oleic acid, linoleic acid, and linolenic acid [7]. The crude fiber in strawberries was extremely low, and in some samples, it was not detected. According to Hotchkiss et al. [29], the soluble fiber identified in strawberry pomace includes pectin, xyloglucan, xylan, β‐glucan, and glucomannan polysaccharides.

#### 2.2.4 Color Parameters (L, a, b*)

Color analysis revealed distinct differences in lightness (L*), redness (a*), and yellowness (b*) among strawberry samples from the four agroclimatic regions (Table 2). Tawangmangu and Ciwidey recorded the highest L* values (35.42 ± 0.08 and 33.71 ± 3.56, respectively), indicating brighter fruit surfaces. Cooler temperatures and higher moisture levels have been reported to support anthocyanin stability and influence cuticle hydration, which may contribute to increased surface reflectance. Recent studies also highlight that fruit brightness could reflect by pigment concentration, epidermal cell morphology, and cuticle composition, suggesting that multiple biochemical and structural factors interact to determine L* values rather than a single environmental determinant [30, 31]. In contrast, Batu and Purbalingga displayed lower L* values (27.31 ± 2.60 and 25.68 ± 3.33), suggesting darker pigmentation possibly linked to warmer climates and higher mineral concentrations.

The a* values, representing the intensity of redness, were highest in Batu (31.52 ± 3.18), followed by Ciwidey (28.28 ± 1.36), indicating stronger anthocyanin accumulation in these regions. This observation appears to be in line with Duan et al. [27], who reported that moderate temperature and light exposure enhance the biosynthesis of pelargonidin derivatives, contributing to red pigmentation. The b* values, representing yellowness, were highest in Ciwidey (11.20 ± 1.96), suggesting a broader spectrum of carotenoid and flavonoid compounds. Purbalingga and Batu exhibited lower b* values (5.22 ± 1.49 and 2.82 ± 1.53), which may indicate limited pigment diversity or faster degradation under warmer conditions. Overall, the color variation across regions suggests possible interactions between agroclimatic factors and soil composition on pigment biosynthesis and stability, both of which are relevant for consumer appeal and antioxidant potential.

#### 2.2.5 pH and Antioxidant Activity

Strawberry samples showed variation in pH and antioxidant activity across the four agroclimatic regions. The highest pH value was recorded in Batu (4.82 ± 0.07), followed by Ciwidey (4.58 ± 0.06), Tawangmangu (4.12 ± 0.07), and Purbalingga (3.58 ± 0.07), indicating that strawberries from Batu and Ciwidey were less acidic and potentially more palatable to consumers. This trend corresponds with their higher sugar content and TSS, suggesting a more favorable sugar‐to‐acid ratio. Conversely, the lower pH in Purbalingga and Tawangmangu may contribute to elevated organic acid biosynthesis, which may be associated with cooler climates and higher soil moisture [27].

Antioxidant activity, measured through DPPH radical scavenging and FRAP (Ferric Reducing Antioxidant Power) assays, was highest in Batu and Ciwidey (96.66 ± 0.16% and 95.94 ± 1.41%, respectively), indicating free radical neutralization capacity. These results are consistent with the presence of anthocyanins and phenolic compounds. Interestingly, Tawangmangu, with its cooler climate and high soil organic matter, showed slightly lower DPPH activity (87.78 ± 1.71%) but the highest TPC (7.41 ± 1.20 mg GAE/100 g), suggesting that antioxidant mechanisms vary by compound type and environmental stress. This apparent discrepancy highlights that antioxidant capacity is not solely determined by total phenolic concentration but also by the structural diversity and reactivity of individual compounds. As reported by Rueangsri et al. [32], phenolic fractions differ in their radical scavenging efficiency depending on extraction methods and compound profiles. Thus, the high phenolic levels in Tawangmangu may include compounds with lower DPPH reactivity, reflecting the complexity of antioxidant mechanisms in strawberries. FRAP values were relatively stable across regions, ranging from 12.19 to 12.99, reflecting consistent ferric ion–reducing capacity. These findings appears to be in line with Sharma et al. [19], who observed that organic fertilization and agroclimatic variation may associated with the antioxidant profiles of strawberries.

This consistency may be associated with the baseline presence of phenolic compounds and organic acids that contribute to ferric ion reduction. Comparable FRAP values (11.8–13.2) have been reported in strawberries cultivated in subtropical Thailand, indicating that strawberries grown under organic fertilization and moderate ecological stress can exhibit strong antioxidant potential [33]. The slightly higher FRAP value recorded in Tawangmangu (12.99 ± 0.05) may be linked to its higher TPC and cooler climate, which may favor the biosynthesis of redox‐active compounds. Although DPPH radical scavenging activity varied more widely across regions, FRAP remained relatively uniform, reinforcing the idea that different antioxidant assays capture distinct mechanisms. FRAP measures electron‐donating capacity, whereas DPPH evaluates hydrogen atom transfer, and both are influenced by compound structure and environmental factors [27].

#### 2.2.6 Total Phenolic Content (TPC)

Total phenolic content ranged from 2.04 to 7.41 mg GAE/100 g (Table 2). Samples from Tawangmangu showed the highest measured levels of phenolic compounds among the four regions studied. The results are in line with the bioactive compounds detected using LC‐HRMS methods, which suggests that strawberries from Tawangmangu may contain a more diverse set of bioactive compounds compared to other samples. Recently, Aksic et al. [34] also reported that TPC for strawberries from Serbia ranged from 1470 to 1700 mg GAE/kg, much higher than from Indonesia. Strawberries from Italy have TPC around 8.05 mg GAE/g dry weight [35]. Furthermore, the TPC of the strawberries has been reported to be influenced by variety, cultivation methods, type of fertilizer, and the agroclimatic conditions [34].

#### 2.2.7 Total Anthocyanin Content (TAC)

Anthocyanin are the major pigment contributing to strawberries coloration (Table 2). Their levels ranged from 5.52 to 18.15 mg cyanidin‐3‐O‐glucoside/ 100 g, with the highest values observed in Batu and the lowest in Purbalingga. Anthocyanin stability is influenced by pH [6] and tend to be more stable under acidic conditions [36]. The color of anthocyanin also varies with acidity [37]. As glycosides, anthocyanins have been reported to exert antioxidant, anticancer, and anti‐inflammatory activities, with some study suggesting potential roles in diabetes management [38]. High anthocyanin levels in strawberries from Batu were consistent with the highest redness (a*). Although anthocyanins are generally more stable under acidic conditions (pH 1–3) [39], the elevated levels observed in Batu may reflect cultivar‐specific traits and agroclimatic influences such as light exposure and nutrient status, rather than pH alone [40]. This indicates that multiple interacting factors may contribute to anthocyanin accumulation and apparent stability in strawberries.

The variation in TAC and fruit color parameters observed in this study also parallels other findings [27], which show that environmental factors such as temperature and light intensity have been reported to be associated with the expression of key enzymes, including chalcone synthase (CHS) and dihydroflavonol 4‐reductase, in anthocyanin biosynthesis. In this study, Ciwidey and Batu, which have moderate to warm temperatures, produced strawberries with higher sugar content and anthocyanin levels, which may be associated with mineral‐rich soils and pigment accumulation. These comparative insights illustrate the potential agroclimatic sensitivity of strawberry phytochemistry and suggest the relevance of region‐specific cultivation strategies to optimize nutritional and functional quality.

#### 2.2.8 Comprehensive Analysis of Strawberry Physicochemical Properties

Titratable acidity and pH values revealed the expected inverse relationship, with Purbalingga showing the lowest titratable acidity (2.84 ± 1.18%) accompanied by the lowest pH (3.58 ± 0.07), whereas Batu exhibited the lowest acidity (4.91 ± 1.61%) together with the highest pH (4.82 ± 0.07) (Table 2). This pattern is consistent with the chemical principle that higher acidity is associated with lower pH values. These differences may be associated with regional variation in organic acid biosynthesis, potentially related to temperature and soil moisture, as noted by Duan et al. [27]. Color parameters further distinguished the samples, with Ciwidey and Tawangmangu exhibiting higher L* and b* values, indicating brighter and more yellowish hues. In contrast, Batu showed the highest a* value (31.52 ± 3.18), consistent with anthocyanin accumulation and more intense red pigmentation.

Antioxidant activity, assessed through DPPH and FRAP assays, was highest in Batu and Ciwidey, was associated with elevated TAC and TPC. Interestingly, Tawangmangu exhibited the highest TPC (7.41 ± 1.20 mg GAE/100 g) despite moderate DPPH activity, suggesting that antioxidant mechanisms may differ by compound type and environmental stress response. Anthocyanin concentration peaked in Batu (18.15 ± 1.11 mg cyanidin‐3‐O‐glucoside/100 g), emphasizing potential influence of agroclimatic conditions on pigment biosynthesis. These findings are consistent with Sharma et al. [19], who highlighted the synergistic effects of organic fertilization and ecological variation on strawberry antioxidant profiles. Overall, the results suggest the relevance of site‐specific agroclimatic and soil factors in shaping the nutritional and functional quality of strawberries. Regions such as Batu and Ciwidey may be favorable for producing sweeter, more pigmented fruits, whereas Tawangmangu may offer potential for cultivating antioxidant‐rich varieties suitable for functional food development.

Spearman's rho analysis revealed several indicative associations between anthocyanin content and agroclimatic factors (Figure 1). Positive correlations were observed anthocyanin content is positively associated with average temperature (*ρ* = 0.40; *p* = 0.600, *n* = 4), annual rainfall (*ρ* = 0.316, *p* = 0.684, *n* = 4), soil organic carbon (*ρ* = 0.40, *p* = 0.600, *n* = 4), available phosphorus (*ρ* = 0.80, *p* = 0.200, *n* = 4), and exchangeable potassium (*ρ* = 0.632, *p* = 0.368, *n* = 4). These relationships suggest that moderate warmth, sufficient rainfall, and fertile soils rich in organic matter and macronutrients may favor anthocyanin biosynthesis. This pattern is consistent with the findings of Duan et al.  [18], who reported that anthocyanin accumulation in *Fragaria x ananassa* is enhanced under balanced environmental conditions that support carbon assimilation and flavonoid pathway activation. Similarly, Wang et al. [41] observed that optimal light and temperature regimes were associated with the upregulation of structural genes such as CHS, flavanone 3‐hydrolase (F_3_H), and anthocyanin synthase (ANS), thereby increasing anthocyanin content and improving fruit coloration.

**FIGURE 1:**
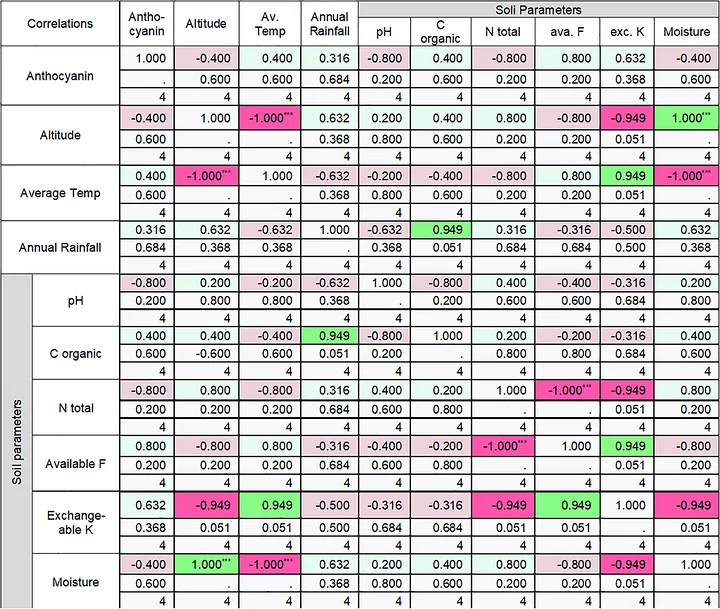
Nonparametric Spearmen rho (ρ) test correlation between anthocyanin content and agroclimatic/soil parameters, with corresponding p‐values (two‐tailed) and sample size (*n* = 4). Positive correlations are shown in green and negative correlations in red, with intensity reflecting the strength of association.

Conversely, negative correlations were found with altitude (*ρ* = −0.40, *p* = 0.600, *n* = 4), soil pH (*ρ* = −0.80, *p* = 0.200, *n* = 4), total nitrogen (*ρ* = −0.80, *p* = 0.200, *n* = 4), and soil moisture (*ρ* = −0.40, *p* = 0.600, *n* = 4) (Figure 1). These inverse relationships suggest that cooler, wetter highland conditions and excessive nitrogen availability may be associated with reduced pigment formation. High altitude typically lowers temperature and solar radiation, which may reduce phenylpropanoid metabolism and anthocyanin synthesis. Elevated soil pH and nitrogen levels can shift nutrient balance toward vegetative growth rather than secondary metabolite production. Lee and Auh [42] reported comparable trends among strawberry cultivars, where excessive nitrogen and alkaline soils diminished anthocyanin concentration and altered metabolite profiles, supporting the hypothesis that nutrient imbalance constrains pigment biosynthesis.

The strong positive intercorrelations among rainfall, organic carbon, and potassium (*ρ* ≈ 0.95, *p* = 0.051, *n* = 4) illustrate the potential association of soil fertility and moisture with the metabolic homeostasis. Rainfall may facilitate organic matter decomposition and nutrient mobility, while potassium may be involved in osmotic regulation and enzyme activation in anthocyanin pathways. However, highly negative correlations such as altitude ↔ temperature (*p*  =  −1.00) and nitrogen ↔ phosphorus (*p*  =  −1.00) suggest that extreme environmental gradients may disrupt nutrient equilibrium and be associated with reduced pigment levels.

Importantly, the small sample size limits the statistical power of these correlations. While some associations reached moderate strength (e.g., *ρ* = 0.80 for anthocyanin and available phosphorus), the corresponding *p*‐values were not consistently below the conventional threshold of 0.05. This underscores the need for caution in interpretation and highlights the importance of expanding future studies to include larger sample sizes and more diverse agroclimatic zones. Overall, these preliminary findings suggest that anthocyanin content in strawberries may be associated with moderate temperature, rainfall, and balanced soil fertility, while extreme altitude, excessive moisture, and nutrient imbalance appear to coincide with reduced pigment levels. These results provide a useful basis for future research aimed at disentangling the relative contributions of climatic and soil factors to strawberry phytochemistry, ideally through multi‐location trials and controlled experiments.

#### 2.2.9 Total Ion Chromatogram (TIC) Analysis

The Total Ion Chromatogram (TIC) profiles obtained from LC‐HRMS analysis revealed distinct molecular complexity across strawberry samples from the four agroclimatic regions. As illustrated in Figure 2, the TIC of Tawangmangu displayed the highest peak density and signal intensity, suggesting a broader spectrum of ionized compounds and molecular diversity under cooler, high‐altitude conditions. This observation is consistent with the region's of elevated soil organic carbon and nitrogen levels, which may be associated with the accumulation of secondary metabolites.

**FIGURE 2:**
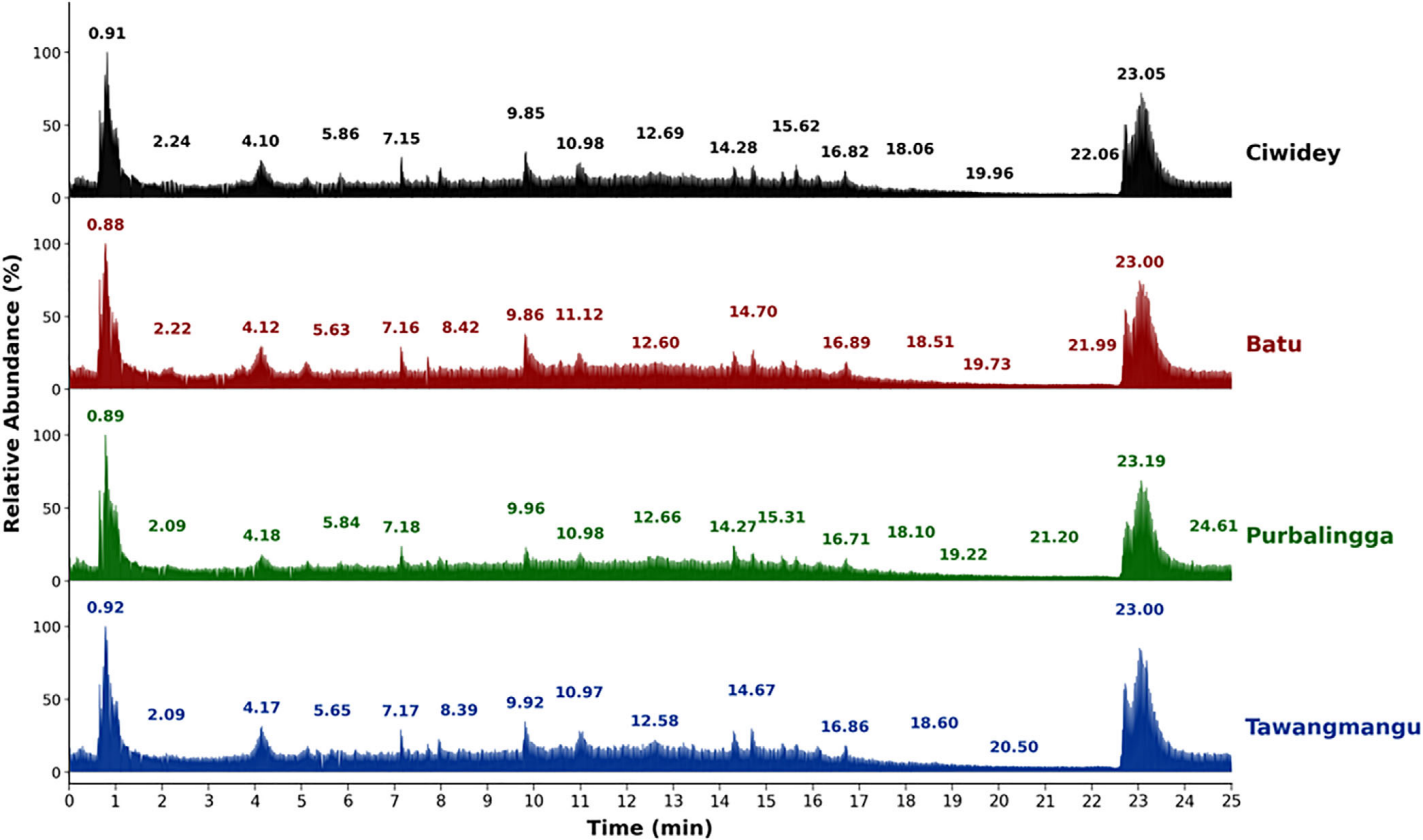
The Total Ion Chromatogram (TIC) of the strawberry from four different agroclimatic regions. The chromatograms illustrate differences in peak density and distribution, reflecting the relative molecular complexity of each sample but not directly representing biosynthetic activity or absolute compound abundance.

In contrast, the TIC profiles of Batu and Purbalingga showed fewer and sharper peaks, reflecting a simpler compound matrix dominated by low‐mass organic acids and volatiles. Ciwidey presented an intermediate profile, showing moderate peak distribution and several high‐m/z signals corresponding to flavonoid conjugates such as miquelianin and kaempferol derivatives. These visible differences in TIC patterns support the LC‐HRMS findings that agroclimatic variation is associated with differences in presence, diversity, and abundance of bioactive compounds. The TIC data serve as molecular fingerprints for each region, suggesting that environmental gradients may modulate strawberry phytochemistry at the ion level. Such chromatographic evidence underscores the relevance of region‐specific cultivation and postharvest management strategies to optimize the retention of functional compounds.

#### 2.2.10 LC‐HRMS Profiling of Bioactive Compounds

The LC‐HRMS analysis revealed distinct bioactive compound profiles in strawberries cultivated across the four agroclimatic regions, underscoring the influence of environmental conditions on phytochemical diversity. Tawangmangu exhibited the most complex and diverse compound spectrum (Table 3), including (Z)‐aconitic acid, GABA, kaempferol, ellagic acid, and conjugated flavonoids such as miquelianin. This richness appears to be associated with cooler climate, higher altitude, and elevated soil organic matter, factors that have been reported to influence secondary metabolite profiles. The presence of GABA and aconitic acid may reflect stress‑related metabolic activity, consistent with Duan et al. [27]. However, the relative contribution of each environmental factor requires further statistical validation.

**TABLE 3:** Organic compounds in strawberries from four different agroclimatic regions.

Compounds	Characteristics of the compound				Relative area (%)			
	Formula	RT (min)	Ionization mode	m/z ratio	CWD	PBL	BAT	TWM
Citric acid	C_6_H_8_O_7_	0.856	[M−H]−	191.0192	64.07	67.87	51.48	52.05
4‐Coumaric acid	C_9_H_8_O_3_	3.752/ 5.230	[M−H]−/[M+H] +	147.0439/ 433.1108	7.01	2.46	14.77	8.79
Cinnamic acid	C_9_H_8_O_2_	5.848	[M+H‐H_2_O] +	131.0491	1.42	1.45	N/A	0.50
Kaempferol	C_15_H_10_O_6_	6.200	[M+H] +	287.0548	0.35	0.29	0.34	0.78
Cyanidanol	C_15_H_8_O_2_	3.022	[M+H] +	291.0861	2.80	2.84	2.37	3.72
Ellagic acid	C_14_H_6_O_8_	5.175	[M+H] +	303.0131	0.59	0.98	0.55	1.45
L‐pyroglutamic acid	C_5_H_7_NO_3_	0.806	[M+H] +	130.0499	0.07	N/A	0.32	N/A
Miquelianin (Quercetin‐3‐O‐glucuronide)	C_21_H_20_O_11_	5.682	[M+H] +	479.0817	8.76	14.19	8.13	6.19
Gamma‐aminobutyric acid (GABA)	C_4_H_9_NO_2_	0.773	[M+H] +	104.0708	3.73	0.99	4.87	13.21
Aromadendrin	C_15_H_12_O_6_	4.828	[M+H] +	289.0704	0.52	0.29	0.34	1.87
Gentiopicrin	C_16_H_20_O_9_	5.829	[M−H]−	355.1037	0.34	N/A	0.56	0.80
(Z)‐Aconitic Acid	C_6_H_6_O_6_	0.850	[M−H]−	173.0086	0.34	N/A	0.21	N/A
Trolox	C_14_H_18_O_4_	9.404	[M+H] +	251.1274	3.14	2.01	4.21	5.56
Trans‐3‐indoleacrylic acid	C_11_H_9_NO_2_	2.514	[M+H] +	188.0704	0.38	1.48	N/A	N/A
But‐3‐ynoic acid	C_4_H_4_O_2_	0.838	[M+H] +	85.0286	0.04	0.17	N/A	N/A

Abbreviations: CWD = Ciwidey, PBL = Purbalingga, BAT = Batu, TWM = Tawangmangu. N/A: not detected.

Ciwidey strawberries were characterized by high levels of GABA and miquelianin, suggesting fermentation‐related metabolic shifts or adaptive responses to moderate climatic stress. Although ellagic acid was not detected in Ciwidey samples, the presence of flavonoid glucuronides indicates alternative antioxidant mechanisms. Purbalingga samples showed strong signals for ellagic acid and genistein, both recognized for their potent antioxidant and anticancer properties, despite their lower sugar content and TSS. This supports the hypothesis that cooler and moderately moist environments may favor phenolic biosynthesis over carbohydrate accumulation.

Batu strawberries exhibited a unique profile dominated by simple organic acids such as but‐3‐ynoic acid and cinnamic acid, which contribute to their sharp aroma and intense red pigmentation. The high anthocyanin content and elevated a* values in Batu samples further support the role of warmer temperatures and mineral‐rich soils in enhancing pigment synthesis. Overall, the LC‐HRMS data confirm that agroclimatic variation affects not only the quantity but also the quality and diversity of bioactive compounds in strawberries, offering valuable insights for cultivar selection, functional food development, and region‐specific postharvest strategies.

The LC‐HRMS profiling in this study revealed a diverse range of bioactive compounds, including ellagic acid, kaempferol, GABA, miquelianin, and cinnamic acid, whose presence varied across agroclimatic regions. LC‐HRMS analysis was performed to profile the chemical constituents of strawberry. The values reported in Table 3 represent relative peak areas (% of total ion current) obtained from chromatographic integration. These values are semi‐quantitative and reflect the relative contribution of each compound to the overall chromatographic profile. The peaks do not correspond to absolute concentrations or molar abundances. Therefore, the results are interpreted as indicative of relative distribution patterns rather than precise quantitative levels. These findings are consistent with recent literature emphasizing the phytochemical richness of strawberries and their sensitivity to environmental factors. Newerli‐Guz et al. [6], identified ellagic acid, pelargonidin‐3‐glucoside, and kaempferol as dominant phenolic compounds in strawberries, highlighting their antioxidant and anti‐inflammatory potentials. Similarly, this study detected ellagic acid and kaempferol predominantly in Purbalingga and Tawangmangu samples, supporting the idea that cooler, organic‐rich environments enhance phenolic biosynthesis.

Barkaoui et al. [43] further demonstrated that postharvest treatments such as gamma irradiation can modulate the phenolic profile of strawberries, particularly by increasing flavonoid content and antioxidant activity. Although this study did not include postharvest processing, the naturally higher antioxidant activity observed in Batu and Ciwidey samples, which correlated with anthocyanin and cinnamic acid content, suggests that agroclimatic stressors may similarly induce phenolic accumulation. Cianciosi et al. [44] also emphasized the importance of compounds such as GABA and miquelianin in neuroprotection and vascular health, noting their occurrence in strawberries cultivated under moderate stress conditions. This observation aligns with the present findings in Ciwidey and Tawangmangu, where GABA and miquelianin were detected in higher relative signal, potentially reflecting adaptive metabolic responses to fluctuating temperature and moisture levels.

These comparisons collectively support the conclusion that agroclimatic variation influences the diversity and concentration of bioactive compounds in strawberries. The coexistence of both low‐mass volatiles and high‐mass flavonoids across regions underscores the metabolic adaptability of strawberries and their potential for targeted functional food development based on regional strengths. While the present analysis highlights associations between agroclimatic variation and strawberry phytochemistry, it must be noted that cultivar/genotype, maturity stage at harvest, seasonal timing, and farming practices may also contribute to the observed chemical differences. These factors represent potential confounders that were not fully controlled in this study, and therefore the findings should be interpreted as preliminary trends requiring validation under more standardized conditions.

This research highlights region‑specific differences in strawberry quality traits, suggesting that environmental and nutrient factors may inform cultivation strategies to enhance fruit sweetness or antioxidant content. Furthermore, the consistent use of organic fermented cattle manure across sites reinforces the viability of sustainable fertilization practices that support both yield and nutritional quality. Policymakers are encouraged to promote site‐adapted cultivation models, invest in soil and climate monitoring infrastructure, and support farmer training in postharvest handling to preserve bioactive integrity. Sobari et al. [45] demonstrated that supplemental white light emiting diode (LED) lighting significantly altered physicochemical traits of Japanese cultivars, with cultivar‐specific responses such as increased flavonoid content and enhanced fruit coloration in ‘Sakura’, elevated vitamin C concentration and dry weight in ‘KS75’, and higher total phenolic content in ‘Haruhi’. Related study also reported that extended photoperiod via LED lighting influenced growth and flowering traits, again highlighting the role of genotype in determining fruit quality [46]. Fruit color variation among regions may not be solely attributed to temperature differences but also to variations in light intensity and spectral composition. Light acts as a key environmental signal regulating anthocyanin biosynthesis through photoreceptors such as phytochromes and cryptochromes, influencing both pigment accumulation and stability. Previous studies have demonstrated that supplemental LED lighting, particularly in the blue–red spectrum, enhances anthocyanin and flavonoid content in strawberries, indicating that light quality interacts with temperature and humidity to determine fruit coloration. Compared to these controlled‐environment findings, the field‐based results suggest that agroclimatic variation interacts with cultivar and management practices, reinforcing the need to consider both environmental and genetic factors when interpreting strawberry phytochemistry in tropical regions.

From a scientific standpoint, this study offers a novel integration of agroclimatic, soil, and LC‐HRMS data to explain regional variations in strawberry phytochemistry. It addresses a critical gap in tropical food chemistry literature by illustrating how environmental gradients influence the biosynthesis of compounds such as ellagic acid, kaempferol, and GABA. The study also introduces a replicable framework for linking agroecological descriptors with functional food potential, providing a foundation for future interdisciplinary research in plant metabolomics, climate‐resilient agriculture, and nutraceutical development.

## 3 Conclusions

This study demonstrates that variations in agroclimatic conditions and soil characteristics may contribute to the strawberry quality across four highland regions of Indonesia. Tawangmangu and Ciwidey showed higher TPC (7.41 ± 1.20 and 6.85 ± 0.95 mg GAE/100 g, respectively) and stronger antioxidant activity (DPPH > 95%), consistent with cooler temperatures and higher moisture levels. In contrast, Batu and Purbalingga exhibited distinct physicochemical profiles, with Batu recording the highest sugar content (2.17 ± 0.31%) and pH (4.82 ± 0.07), while Ciwidey showed the greatest titratable acidity (5.68 ± 0.90%). These quantitative differences highlight region‑specific influences on fruit composition. The LC‐HRMS analysis confirmed the presence of diverse bioactive compounds, including ellagic acid, kaempferol, GABA, and miquelianin, whose distribution was closely related to environmental conditions and soil mineral composition.

These findings underscore the metabolic plasticity of strawberries and highlight the importance of site‐specific cultivation strategies to optimize nutrition and functional quality. The combination of physicochemical assays and LC‐HRMS profiling provides a comprehensive framework to understand region‐specific fruit characteristics. In summary, the present study provides preliminary insights into the potential influence of agroclimatic variation on strawberry phytochemistry across four highland regions. However, given the limited number of locations and the possible confounding effects of cultivar, harvest maturity, seasonal timing, and farming practices, these findings should be regarded as indicative trends rather than generalized conclusions. Future research with broader sampling and controlled experimental conditions will be necessary to establish more definitive relationships.

## 4 Experimental Section

### 4.1 Soil Sampling Methods

Soil samples were collected from four cultivation sites in Java, Indonesia: Tawangmangu (Karanganyar Regency, Central Java Province), Batu (East Java Province), Ciwidey (West Java Province), and Purbalingga (Central Java Province). Agroclimatic data (temperature and rainfall) were obtained from the Indonesian Agency for Meteorology, Climatology, and Geophysics (BMKG‐Badan Meteorologi, Klimatologi, dan Geofisika) through regional stations, and supplemented by district‐level agricultural extension service reports. The extension data provide mean values with seasonal intervals, while farmer interviews were used to confirm consistency of cultivation conditions during the sampling period. Farmer respondents were selected purposively based on their experience in strawberry cultivation. Each respondent had a minimum of 5 years of continuous farming experience in the respective region, ensuring that the agroclimatic and cultivation data obtained through interviews represented reliable and consistent local practices.

To complement the agroclimatic assessment, soil samples were collected from each strawberry cultivation site to evaluate baseline soil fertility and its potential influence on fruit quality. Composite samples were taken from the root zone (0–20 cm depth) using a stainless‐steel auger as this layer contains the highest concentration of organic matter and available nutrients and is most directly influenced by agroclimatic conditions and cultivation practices [47]. Each cultivation field had an approximate size of at least 750 m^2^, managed by smallholder farmers. Within each field, five subsamples were systematically taken from the four corners and the center of the plot, then homogenized to form one composite sample per site. This grid‐based approach was adopted to capture spatial variability and improve reproducibility of soil analysis. Soil properties such as pH, organic matter, and nutrient availability can vary considerably within a single field due to micro‑environmental differences, management practices, and slope conditions. By sampling multiple points, the study reduces bias from localized anomalies and provides a more reliable characterization of the soil environment influencing strawberry growth. This approach follows standard agronomic protocols, which recommend composite sampling from several spots within a plot to improve accuracy and reproducibility of soil analysis [48].

The sample were air‐dried, homogenized, and sieved through a 2 mm mesh before analysis. Soil pH was measured in a 1:2.5 soil‐to‐water suspension using a calibrated pH meter. Prior to soil pH determination, the pH meter was calibrated using standard buffer solutions at pH 4.0, 7.0, and 9.0 to ensure accuracy across the measurement range. Calibration was performed daily before sample analysis, following manufacturer guidelines, and electrodes were rinsed thoroughly with distilled water between buffer solutions to prevent cross‑contamination. All soil analyses were conducted in triplicate to ensure reproducibility. Soil pH, organic carbon, total nitrogen, available phosphorus, and exchangeable potassium were determined using standard methods (Walkley‐Black, Kjeldahl, Bray I, Olsen, and flame photometry).

### 4.2 Parametric Analysis of Soil

Organic carbon (C) content was determined via the Walkley‐Black method [48, 49], while total nitrogen (N) was analyzed using the Kjeldahl method [50]. Available phosphorus (P) was quantified using the Bray I extraction followed by spectrophotometric detection, and potassium (K) was measured using flame photometry [50, 51]. Exchangeable potassium was expressed as milliequivalents per 100 g soil (meq/100 g), equivalent to cmol (+)/kg soil, based on calibration with KCl standards. Soil moisture content was determined gravimetrically by oven‐drying at 105°C until a constant weight was achieved. These parameters were used to characterize soil fertility and support the interpretation of the physicochemical and bioactive profiles of strawberries across different agroclimatic zones [52]. Available phosphorus (P) content (ppm) was also analyzed using the Olsen method [53]. The Bray I method was applied for soils with acidic pH (<6.5), as it is widely recommended for such conditions, while the Olsen method was used for soils with neutral to slightly alkaline pH (>6.5). This dual approach ensures that phosphorus availability is accurately assessed according to the specific agroclimatic and soil characteristics of each cultivation site [54].

### 4.3 Strawberry Sampling

Strawberry samples were collected from four highland regions in Java, Indonesia: Batu (East Java Province), Ciwidey (West Java Province), Purbalingga (Central Java Province), and Tawangmangu (Karanganyar regency, Central Java Province). These locations were selected based on their distinct agroclimatic characteristics, known to influence fruit development and bioactive compound accumulation. The cultivation sites were located at altitudes ranging from 1200 m to 1800 m above sea level. Batu and Ciwidey are situated between 1200 m and 1700 m; while Tawangmangu and Purbalingga reach up to 1800 m. Average annual temperatures ranged from 15°C to 22°C, with cooler conditions observed at higher elevations. Annual rainfall varied from 2000 mm to 3500 mm, and soil types included volcanic loam (Ciwidey and Tawangmangu), sandy clay (Purbalingga), and fertile andosol (Batu). These environmental parameters were obtained from local agricultural extension data and farmer interviews and were used to contextualize the physicochemical and bioactive profiles of the strawberry.

Fresh strawberry fruits were collected directly from local farmers during the peak harvest season to ensure optimal ripeness. Voucher specimens of strawberry (Fragaria x ananassa Duch., cv Mencir) were identified by researcher from Research Center for Technology and Food Processing, BRIN, collected from Ciwidey, Bandung, West Java, Indonesia (7.1135°S, 107.4431°E) in June 2025. The specimens were deposited at Herbarium Bogoriense (BO) Indonesian Institute of Sciences, under provisional registration code‚ BRIN‐STRAW‐MENCIR‐2025.'' At each location, fruits were selected based on uniform maturity (≥80% surface redness and weigh range was 8–12 g per fruit.), absence of physical damage, and standard size (neither undersized nor oversized). A minimum of 2 kg of strawberries per site was collected, comprising five biological replicates pooled from different plots and the same farm to represent regional variability. The fruits were homogenized to obtain a composite sample, which was then subjected to laboratory analysis. Each chemical parameter was measured in triplicate (technical replicates) to ensure reproducibility and accuracy.

Immediately after collection, fruits were rinsed with potable water to remove surface debris and stored at −20°C until physicochemical and bioactive compound analyses were conducted (maximum for 5 days). Freezing at −20°C is a widely accepted practice in fruit quality studies, as confirmed by previous reports [53, 55], which demonstrate that low‑temperature storage effectively stabilizes bioactive compounds and prevents spoilage. To reduce potential postharvest changes and ensure comparability across agroclimatic zones, samples were collected following standardized handling procedures. Fruits were harvested at uniform maturity, placed in clean containers, and transported under cooled conditions to the laboratory within a few hours of collection. These measures are consistent with internationally recognized postharvest handling practices, which have been shown to slow physiological and biochemical deterioration in fresh produce and improve the reliability of subsequent chemical analyses [56].

At all cultivation sites, strawberries were grown using traditional organic fertilization practices, primarily manure‐based fertilizers, which were practiced by farmers in the highland regions of Java. This fertilizer, derived from decomposed livestock waste, enriches the soil by enhancing organic matter content, microbial activity, and nutrient availability, particularly nitrogen, phosphorus, and potassium. The use of manure represents a low‐input, sustainable farming approach that may affect the accumulation of bioactive compounds in strawberry fruits. Although fertilization rates were not standardized across sites, this condition is acknowledged as a methodological limitation, the consistent use of organic inputs provides a shared baseline for assessing the influence of agroclimatic variation on fruit quality. This variability in manure application reflects real farming practices and should be considered when interpreting site‐specific differences.

### 4.4 Physicochemical Analysis

The physicochemical analysis of fresh strawberries included measurements of moisture, fat, protein, dietary fiber, and total carbohydrates [28], as well as color parameters (L*, a*, and b*), total soluble solids (TSS), total phenolic content (TPC), total anthocyanin content (TAC) [21], pH, and antioxidant activity (% RSA DPPH and FRAP value). All analysis were performed in three replicates except for color measurement. The DPPH radical scavenging activity was determined using a 0.1 mM DPPH solution in methanol, without the use of an external antioxidant standard. The values reported represent the percentage of radical scavenging activity calculated relative to the blank (aquadest) absorbance, based on triplicate measurements. The total phenolic content was determined using the Folin–Ciocalteu method [21] with minor modifications.

### 4.5 Color Analysis

Fruit color was measured using a Chroma Meter (CR‐400, Konica Minolta, Japan) in reflectance mode. For each sampling location, 10 representative fruits were selected. On each fruit, color was measured at three different points on the surface (apical, equatorial, and basal regions). The L*, a*, and b* values obtained from these three points were averaged to represent the color parameters of each fruit. Subsequently, the averaged values from the 10 fruits were used to calculate the mean color parameters for each location.

### 4.6 Sample Preparation for TPC, TAC, and Antioxidant Activity

Extracts were prepared by macerating 25 g of fresh fruits using 25 mL of 70% of methanol and left for an hour. The ethanol extract was filtered and used as a stock solution for further analysis. For TPC analysis, a 0.5 mL extract was mixed with 2.5 mL of Folin–Ciocalteu reagent (diluted with water at a 1:10 ratio) and 2 mL of sodium carbonate solution (7.5%). After incubation at room temperature for 30 min, absorbance was measured at 765 nm using a UV–VIS spectrophotometer (Shimadzu UV‐1800, Japan). Results were expressed as mg gallic acid equivalents (GAE) per 100 g of fresh weight. Gallic acid with the concentration of 1 mg/mL was used as a standard.

The TAC was measured using the pH differential method [57]. The extract was diluted in buffers at pH 1 and 4.5, and the absorbance was measured at 520 and 700 nm. Anthocyanin concentration (mg cyanidin‐3‐glucoside equivalents per 100 g) was calculated using a molar extinction coefficient of 26,900 L mol^−^^1^ cm^−^^1^. TAC was determined spectrophotometrically using quartz cuvettes with a fixed path length of 1 cm.

Antioxidant activity based on DPPH radical scavenging was determined by preparing a 0.1 mM DPPH solution in methanol, mixing it with the sample, and incubating the mixture in the dark for 30 min [15]. Absorbance was measured at 517 nm [16] with minor modifications, and the radical scavenging activity was calculated as follows:

(1) $$\%\mathrm{RSA}=\left[\left(A\_\mathrm{control}-A\_\mathrm{sample}\right)/A\_\mathrm{control}\right]\times 100$$

Antioxidant activity was also determined using the Ferric Reducing Antioxidant Power (FRAP) method [17]. The FRAP reagent was prepared by mixing 300 mM acetate buffer, 10 mM TPTZ solution in 40 mM hydrochloric acid, and 20 mM FeCl_3_·6H_2_O solution. The sample was reacted with the reagent solution and incubated for 30 min at 37°C. The absorbance of the Fe^2^^+^‐TPTZ complex was measured at 593 nm against blanks using a UV–VIS spectrophotometer.

### 4.7 LC‐HRMS Analysis

The LC–HRMS analysis was conducted using a Thermo Accela HPLC (high performance liquid chromatography) system (Thermo Fisher Scientific, San Jose, CA, USA) coupled with a Thermo Instruments MS system (Finnigan LTQ/LTQ Orbitrap). Gradient elution was performed on a Poroshell EC‐C18 RP analytical HPLC column (2.7 µm, 2.1 × 100 mm, Agilent, USA), applying a mobile phase of 0–100% MeCN for 25 min, followed by 100% MeCN for 5 min at a flow rate of 0.5 mL min^−^^1^. The capillary temperature and voltage were maintained at 260°C and 45 V, respectively. Auxiliary gas flow was set at 10–20 arbitrary units, sheath gas flow at 40–50 arbitrary units, and spray voltage at 4.5 kV. Full‐scan mass spectra were recorded over a range of 100–2000 m/z (maximum resolution 60 000). The sample injection volume was 20 µL. The database used for LC‑HRMS interpretation was derived from publicly available metabolite libraries (mzCloud and ChemSpider), which provide exact protonated monoisotopic masses, retention times, and isotopic patterns. Compound identification was based on accurate mass determination, retention time comparison, and isotopic pattern similarity. Validation was performed by comparing MS/MS fragmentation patterns of selected analytes with reference spectra available in these libraries, ensuring reliable identification of bioactive compounds in strawberry extracts.

### 4.8 Statistical Analysis

All data were expressed as mean ± standard deviation. One‐way ANOVA was performed to evaluate differences among agroclimatic regions, with significance set at α = 0.05. The Number Cruncher Statistical System (NCSS) software, Statistical and Graphics Analysis (NCSS, LLC, USA, version 2020), was used for variance comparison. This study did not involve human or animal subjects; therefore, sex and gender‐based analyses (SGBA) were not applicable.

To evaluate the relationships among multiple agroclimatic and soil parameters and their influence on strawberry anthocyanin content, a nonparametric Spearman's rank correlation (ρ) test was employed. Spearman's rho assesses the strength and direction of monotonic associations between ranked variables, allowing detection of both linear and nonlinear trends. The analysis was conducted across all agroclimatic factors (altitude, average temperature, annual rainfall) and soil properties (pH, organic carbon, total nitrogen, available phosphorus, exchangeable potassium, and moisture content). Correlation coefficients were interpreted as follows: *ρ* ≥ 0.70 indicated a strong relationship, 0.40–0.69 moderate, and <0.40 weak. Significance levels were set at *p* < 0.05 and *p* < 0.01. The resulting correlation matrix and heatmap visualization were used to identify multifactor interactions that potentially regulate anthocyanin biosynthesis under varying environmental conditions.

## Author Contributions

**Soraya Habibi**: conceptualization, methodology, data curation, validation, supervision, funding acquisition, project administration, resources, writing – original draft, writing – review and editing. **Einstivina Nuryandani**: conceptualization, methodology, data curation, supervision, funding acquisition, project administration, resources, writing – original draft, writing – review and editing. **Nanik Suhartatik**: conceptualization, methodology, data curation, data validation, visualization, writing – original draft, writing – review and editing. **Fadilah Husnun**: methodology, data curation, validation, formal analysis, supervision, writing – review and editing. **Akhmad Mustofa**: methodology, software, data curation, validation, supervision, visualization, writing – review and editing. **Nurika Yepi Prasetyaningtyas**: conceptualization, methodology, data curation, formal analysis, visualization, writing – original draft. **Titiek Farianti Djaafar**: conceptualization, methodology, data curation, investigation, validation, formal analysis, supervision, visualization, resources, writing – original draft, writing – review and editing. **Tri Marwati**: conceptualization, methodology, data curation, investigation, validation, formal analysis, supervision, resources, writing – original draft, writing – review and editing. **Anna Fajariyah**: conceptualization, methodology, data curation, investigation, validation, formal analysis, supervision, visualization, resources, writing – original draft, writing – review and editing. **Ulyatu Fitrotin**: conceptualization, methodology, data curation, validation, formal analysis, visualization, writing – original draft, writing – review and editing

## Funding

This study was supported by Universitas Terbuka, Kementerian Riset dan Teknologi/Badan Riset dan Inovasi Nasional, and Universitas Slamet Riyadi.

## Conflicts of Interest

The authors declare no conflicts of interest.

## Declaration of Generative AI in Scientific Writing

During the preparation of this manuscript, the authors used tools such as Perplexity and Connected Papers to explore related literature and identify correlated articles. Grammarly and QuillBot were employed for grammar checking. Portions of the text were refined with the assistance of Microsoft Copilot AI, under the supervision of the authors, who reviewed and approved all content and take full responsibility for the published work.

## Data Availability

Data available on request from the authors.
